# Bis(3-meth­oxy-6-methyl-2-pyrid­yl) ether

**DOI:** 10.1107/S160053680706775X

**Published:** 2008-01-04

**Authors:** Yuan-Yuan Jiang, Hong-Hong Lan, Dong-Sheng Deng, Bao-Ming Ji

**Affiliations:** a College of Chemistry and Molecular Engineering, Qingdao University of Science and Technology, Qingdao 266042, People’s Republic of China; b Department of Chemistry, Zhengzhou University, Zhengzhou 450052, People’s Republic of China; c College of Chemistry and Chemical Engineering, Luoyang Normal University, Luoyang 471022, People’s Republic of China

## Abstract

In the mol­ecule of the title compound, C_14_H_16_N_2_O_3_, the dihedral angle between the pyridyl rings is 87.74 (3)°. In the crystal structure, inter­molecular C—H⋯O hydrogen bonds link the mol­ecules into infinite zigzag chains.

## Related literature

For related literature, see: Jung *et al.* (1997 ▶); Dunne *et al.* (1995 ▶); Wang *et al.* (2001 ▶); Goulle *et al.* (1993 ▶); Gilat *et al.* (1995 ▶); Kawai *et al.* (1995 ▶); Gütlich *et al.* (1994 ▶). For bond-length data, see: Allen *et al.* (1987 ▶).
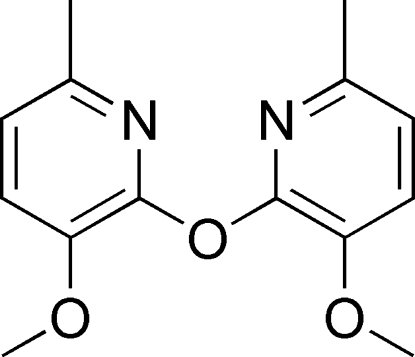


## Experimental

### 

#### Crystal data

C_14_H_16_N_2_O_3_
*M* *_r_* = 260.29
Monoclinic, 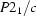

*a* = 12.146 (2) Å
*b* = 7.5372 (15) Å
*c* = 14.669 (3) Å
β = 94.577 (3)°
*V* = 1338.6 (4) Å^3^
*Z* = 4
Mo *K*α radiation
μ = 0.09 mm^−1^
*T* = 294 (2) K
0.29 × 0.21 × 0.13 mm

#### Data collection

Bruker SMART CCD area-detector diffractometer
Absorption correction: multi-scan (*SADABS*; Sheldrick, 1996 ▶) *T*_min_ = 0.974, *T*_max_ = 0.988
8226 measured reflections
2477 independent reflections
1229 reflections with *I* > 2σ(*I*)
*R*_int_ = 0.041

#### Refinement

*R*[*F* ^2^ > 2σ(*F* ^2^)] = 0.048
*wR*(*F* ^2^) = 0.146
*S* = 1.01
2477 reflections
177 parameters
H-atom parameters constrained
Δρ_max_ = 0.13 e Å^−3^
Δρ_min_ = −0.11 e Å^−3^

### 

Data collection: *SMART* (Bruker, 2004 ▶); cell refinement: *SAINT* (Bruker, 2004 ▶); data reduction: *SAINT*; program(s) used to solve structure: *SHELXS97* (Sheldrick, 1997 ▶); program(s) used to refine structure: *SHELXL97* (Sheldrick, 1997 ▶); molecular graphics: *SHELXTL* (Bruker, 2004 ▶); software used to prepare material for publication: *SHELXTL*.

## Supplementary Material

Crystal structure: contains datablocks global, I. DOI: 10.1107/S160053680706775X/hk2409sup1.cif

Structure factors: contains datablocks I. DOI: 10.1107/S160053680706775X/hk2409Isup2.hkl

Additional supplementary materials:  crystallographic information; 3D view; checkCIF report

## Figures and Tables

**Table 1:** Hydrogen-bond geometry (Å, °)

*D*—H⋯*A*	*D*—H	H⋯*A*	*D*⋯*A*	*D*—H⋯*A*
C6—H6⋯O3^i^	0.93	2.52	3.358 (3)	150

Symmetry code: (i) 
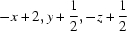
.

**Fig. 1.**
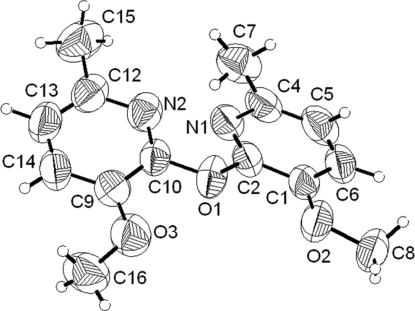
The molecular structure of the title molecule, with the atom-numbering scheme. Displacement ellipsoids are drawn at the 30% probability level.

**Fig. 2.**
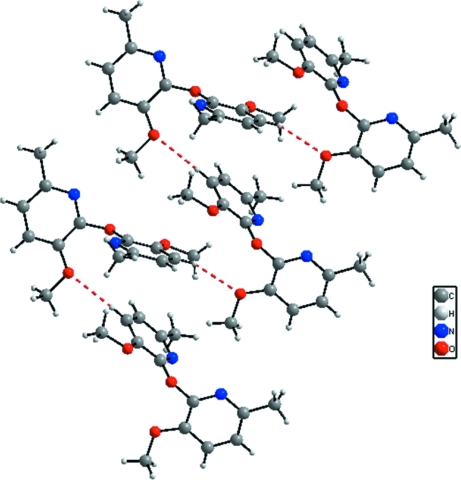
A packing diagram of (I). Hydrogen bonds are shown as dashed lines.

## supplementary crystallographic information

### Comment

2,2'-Dipyridylether and its derivatives are a kind of extensively studied (Jung *et al.*, 1997; Dunne *et al.*, 1995; Wang *et al.*, 2001; Goulle *et al.*, 1993) multifuntional organic ligands. Most research in this area has focused on conjugated organic molecules undergoing frequency-sensitive reversible bond-forming reactions, for the design of inorganic or organometallic switches (Gilat *et al.*, 1995; Kawai *et al.*, 1995; Gütlich *et al.*, 1994). As part of our ongoing studies, we report herein the synthesis and crystal structure of the title compound, (I).

In the molecule of (I) (Fig. 1), the bond lengths (Allen *et al.*, 1987) and angles are within normal ranges. Rings A (N1/C1/C2/C4—C6) and B (N2/C9/C10/C12—C14) are, of course, planar and the dihedral angle between them is A/B = 87.74 (3)°.

In the crystal structure, intermolecular C—H···O hydrogen bonds (Table 1) link the molecules into infinite zigzag chains (Fig. 2), in which they seem to be effective in the stabilization of the structure.

### Experimental

For the preparation of the title compound, 2-iodo-3-methoxy-6-methylpyridine (250 mg, 1 mmol) and active Cu powder (511 mg, 8 mmol) were added to a solution of DMF (10 ml). The resulting mixture was heated at 428 K for 24 h under nitrogen atmosphere. After the active Cu powder was filtered, the filtrate was washed with water (3 × 20 ml), and the aqueous layer was extracted by ethyl acetate (3 × 20 ml). The combined organic layer was dried over anhydrous MgSO_4_, and the solvent was removed *in vacuo* to give the crude product. After purification by silica gel chromatography, a clear solution was set aside to crystallize.

### Refinement

H atoms were positioned geometrically, with C—H = 0.93 and 0.96 Å for aromatic and methyl H, and constrained to ride on their parent atoms, with *U*_iso_(H) = *xU*_eq_(C), where *x* = 1.5 for methyl H, and *x* = 1.2 for aromatic H atoms.

### Crystal data

**Table:**

C_14_H_16_N_2_O_3_	*F*_000_ = 552
*M**_r_* = 260.29	*D*_x_ = 1.292 Mg m^−^^3^
Monoclinic, *P*2_1_/*c*	Mo *K*α radiation λ = 0.71073 Å
Hall symbol: -P 2ybc	Cell parameters from 1190 reflections
*a* = 12.146 (2) Å	θ = 2.8–20.3º
*b* = 7.5372 (15) Å	µ = 0.09 mm^−^^1^
*c* = 14.669 (3) Å	*T* = 294 (2) K
β = 94.577 (3)º	Block, colorless
*V* = 1338.6 (4) Å^3^	0.29 × 0.21 × 0.13 mm
*Z* = 4	

### Data collection

**Table:**

Bruker SMART CCD area-detector diffractometer	2477 independent reflections
Radiation source: fine-focus sealed tube	1229 reflections with *I* > 2σ(*I*)
Monochromator: graphite	*R*_int_ = 0.041
*T* = 294(2) K	θ_max_ = 25.5º
φ and ω scans	θ_min_ = 2.8º
Absorption correction: multi-scan(SADABS; Sheldrick, 1996)	*h* = −14→14
*T*_min_ = 0.974, *T*_max_ = 0.988	*k* = −9→8
8226 measured reflections	*l* = −17→17

### Refinement

**Table:**

Refinement on *F*^2^	Hydrogen site location: inferred from neighbouring sites
Least-squares matrix: full	H-atom parameters constrained
*R*[*F*^2^ > 2σ(*F*^2^)] = 0.048	*w* = 1/[σ^2^(*F*_o_^2^) + (0.0653*P*)^2^ + 0.0571*P*] where *P* = (*F*_o_^2^ + 2*F*_c_^2^)/3
*wR*(*F*^2^) = 0.147	(Δ/σ)_max_ < 0.001
*S* = 1.01	Δρ_max_ = 0.13 e Å^−^^3^
2477 reflections	Δρ_min_ = −0.11 e Å^−^^3^
177 parameters	Extinction correction: SHELXL97 (Sheldrick, 1997), Fc^*^=kFc[1+0.001xFc^2^λ^3^/sin(2θ)]^-1/4^
Primary atom site location: structure-invariant direct methods	Extinction coefficient: 0.0071 (19)
Secondary atom site location: difference Fourier map	

### Special details

**Table:**

Geometry. All e.s.d.'s (except the e.s.d. in the dihedral angle between two l.s. planes) are estimated using the full covariance matrix. The cell e.s.d.'s are taken into account individually in the estimation of e.s.d.'s in distances, angles and torsion angles; correlations between e.s.d.'s in cell parameters are only used when they are defined by crystal symmetry. An approximate (isotropic) treatment of cell e.s.d.'s is used for estimating e.s.d.'s involving l.s. planes.
Refinement. Refinement of *F*^2^ against ALL reflections. The weighted *R*-factor *wR* and goodness of fit *S* are based on *F*^2^, conventional *R*-factors *R* are based on *F*, with *F* set to zero for negative *F*^2^. The threshold expression of *F*^2^ > σ(*F*^2^) is used only for calculating *R*-factors(gt) *etc*. and is not relevant to the choice of reflections for refinement. *R*-factors based on *F*^2^ are statistically about twice as large as those based on *F*, and *R*- factors based on ALL data will be even larger.

### Fractional atomic coordinates and isotropic or equivalent isotropic displacement parameters (Å^2^)

**Table:**

	*x*	*y*	*z*	*U*_iso_*/*U*_eq_	
O1	0.77209 (13)	0.0344 (2)	0.12530 (11)	0.0774 (6)	
O2	0.97934 (14)	0.1001 (2)	0.10596 (15)	0.0854 (6)	
O3	0.72046 (17)	−0.3036 (3)	0.15773 (14)	0.0996 (7)	
N1	0.79139 (16)	0.0722 (3)	0.28290 (16)	0.0669 (6)	
N2	0.59034 (19)	0.1178 (3)	0.11932 (13)	0.0747 (7)	
C1	0.9468 (2)	0.1105 (3)	0.1925 (2)	0.0677 (7)	
C2	0.8363 (2)	0.0727 (3)	0.2043 (2)	0.0630 (7)	
C4	0.8564 (2)	0.1129 (3)	0.3590 (2)	0.0736 (8)	
C5	0.9660 (3)	0.1532 (4)	0.3532 (2)	0.0884 (9)	
H5	1.0099	0.1810	0.4061	0.106*	
C6	1.0115 (2)	0.1529 (3)	0.2703 (2)	0.0837 (9)	
H6	1.0856	0.1813	0.2671	0.100*	
C7	0.8040 (2)	0.1079 (4)	0.44789 (19)	0.0953 (9)	
H7A	0.7319	0.1603	0.4401	0.143*	
H7B	0.8489	0.1731	0.4932	0.143*	
H7C	0.7978	−0.0130	0.4674	0.143*	
C8	1.0953 (2)	0.1180 (4)	0.0964 (2)	0.0986 (10)	
H8A	1.1190	0.2350	0.1148	0.148*	
H8B	1.1096	0.0990	0.0337	0.148*	
H8C	1.1352	0.0318	0.1343	0.148*	
C9	0.6354 (2)	−0.1871 (4)	0.14619 (17)	0.0750 (8)	
C10	0.6628 (2)	−0.0120 (4)	0.13349 (16)	0.0660 (7)	
C12	0.4831 (2)	0.0807 (5)	0.11836 (18)	0.0813 (9)	
C13	0.4500 (3)	−0.0916 (5)	0.1311 (2)	0.0928 (10)	
H13	0.3749	−0.1166	0.1301	0.111*	
C14	0.5245 (3)	−0.2276 (5)	0.14539 (19)	0.0889 (9)	
H14	0.5010	−0.3433	0.1542	0.107*	
C15	0.4036 (2)	0.2307 (5)	0.1025 (2)	0.1091 (11)	
H15A	0.3917	0.2861	0.1598	0.164*	
H15B	0.3347	0.1862	0.0749	0.164*	
H15C	0.4332	0.3160	0.0624	0.164*	
C16	0.6933 (3)	−0.4891 (4)	0.1585 (2)	0.1124 (11)	
H16A	0.6520	−0.5143	0.2100	0.169*	
H16B	0.7601	−0.5579	0.1627	0.169*	
H16C	0.6498	−0.5190	0.1031	0.169*	

### Atomic displacement parameters (Å^2^)

**Table:**

	*U*^11^	*U*^22^	*U*^33^	*U*^12^	*U*^13^	*U*^23^
O1	0.0539 (11)	0.1004 (15)	0.0774 (12)	−0.0163 (10)	0.0011 (9)	0.0035 (10)
O2	0.0556 (12)	0.0860 (14)	0.1149 (16)	−0.0083 (9)	0.0090 (10)	0.0070 (12)
O3	0.0906 (15)	0.0842 (15)	0.1229 (17)	0.0000 (12)	0.0017 (12)	0.0031 (12)
N1	0.0656 (14)	0.0569 (14)	0.0764 (15)	−0.0016 (10)	−0.0059 (12)	0.0004 (11)
N2	0.0645 (15)	0.0939 (18)	0.0648 (14)	−0.0066 (14)	0.0002 (11)	−0.0061 (12)
C1	0.0533 (16)	0.0486 (15)	0.100 (2)	−0.0005 (12)	0.0001 (16)	0.0009 (15)
C2	0.0559 (16)	0.0473 (15)	0.083 (2)	−0.0043 (12)	−0.0124 (15)	0.0040 (14)
C4	0.078 (2)	0.0514 (16)	0.088 (2)	0.0046 (14)	−0.0149 (17)	−0.0074 (14)
C5	0.082 (2)	0.068 (2)	0.108 (3)	−0.0014 (16)	−0.0298 (19)	−0.0185 (18)
C6	0.0581 (17)	0.0625 (19)	0.128 (3)	−0.0084 (14)	−0.0108 (19)	−0.0091 (18)
C7	0.111 (2)	0.087 (2)	0.086 (2)	0.0068 (18)	−0.0066 (18)	−0.0127 (17)
C8	0.0561 (18)	0.091 (2)	0.150 (3)	−0.0090 (16)	0.0203 (17)	0.006 (2)
C9	0.0730 (19)	0.077 (2)	0.0749 (19)	−0.0030 (17)	0.0040 (15)	−0.0077 (16)
C10	0.0541 (16)	0.080 (2)	0.0635 (17)	−0.0112 (15)	0.0030 (12)	−0.0036 (15)
C12	0.064 (2)	0.112 (3)	0.0676 (18)	−0.0007 (18)	0.0036 (14)	−0.0150 (17)
C13	0.0588 (18)	0.127 (3)	0.094 (2)	−0.019 (2)	0.0089 (16)	−0.032 (2)
C14	0.075 (2)	0.099 (2)	0.093 (2)	−0.0320 (19)	0.0139 (17)	−0.0205 (19)
C15	0.079 (2)	0.145 (3)	0.102 (2)	0.030 (2)	−0.0016 (17)	−0.009 (2)
C16	0.146 (3)	0.071 (2)	0.124 (3)	−0.006 (2)	0.037 (2)	0.000 (2)

### Geometric parameters (Å, °)

**Table:**

O1—C2	1.374 (3)	C7—H7C	0.9600
O1—C10	1.388 (3)	C8—H8A	0.9600
O2—C1	1.361 (3)	C8—H8B	0.9600
O2—C8	1.433 (3)	C8—H8C	0.9600
O3—C9	1.356 (3)	C9—C14	1.381 (4)
O3—C16	1.437 (3)	C10—N2	1.321 (3)
N1—C2	1.315 (3)	C10—C9	1.377 (4)
N1—C4	1.350 (3)	C12—C13	1.377 (4)
N2—C12	1.331 (3)	C12—C15	1.493 (4)
C1—C6	1.371 (4)	C13—C14	1.372 (4)
C2—C1	1.396 (3)	C13—H13	0.9300
C4—C5	1.375 (4)	C14—H14	0.9300
C4—C7	1.496 (3)	C15—H15A	0.9600
C5—H5	0.9300	C15—H15B	0.9600
C6—C5	1.376 (4)	C15—H15C	0.9600
C6—H6	0.9300	C16—H16A	0.9600
C7—H7A	0.9600	C16—H16B	0.9600
C7—H7B	0.9600	C16—H16C	0.9600
			
C2—O1—C10	117.5 (2)	H8A—C8—H8C	109.5
C1—O2—C8	116.6 (2)	H8B—C8—H8C	109.5
C9—O3—C16	117.3 (2)	O3—C9—C10	116.6 (2)
C2—N1—C4	118.0 (2)	O3—C9—C14	126.2 (3)
C10—N2—C12	119.0 (3)	C10—C9—C14	117.1 (3)
O2—C1—C6	126.9 (3)	N2—C10—C9	124.5 (2)
O2—C1—C2	117.2 (2)	N2—C10—O1	115.4 (2)
C6—C1—C2	115.9 (3)	C9—C10—O1	119.7 (3)
N1—C2—O1	119.5 (2)	N2—C12—C13	119.6 (3)
N1—C2—C1	125.4 (3)	N2—C12—C15	117.6 (3)
O1—C2—C1	115.1 (3)	C13—C12—C15	122.9 (3)
N1—C4—C5	120.3 (3)	C14—C13—C12	121.9 (3)
N1—C4—C7	117.0 (3)	C14—C13—H13	119.0
C5—C4—C7	122.7 (3)	C12—C13—H13	119.0
C4—C5—C6	120.9 (3)	C13—C14—C9	117.9 (3)
C4—C5—H5	119.5	C13—C14—H14	121.1
C6—C5—H5	119.5	C9—C14—H14	121.1
C1—C6—C5	119.5 (3)	C12—C15—H15A	109.5
C1—C6—H6	120.3	C12—C15—H15B	109.5
C5—C6—H6	120.3	H15A—C15—H15B	109.5
C4—C7—H7A	109.5	C12—C15—H15C	109.5
C4—C7—H7B	109.5	H15A—C15—H15C	109.5
H7A—C7—H7B	109.5	H15B—C15—H15C	109.5
C4—C7—H7C	109.5	O3—C16—H16A	109.5
H7A—C7—H7C	109.5	O3—C16—H16B	109.5
H7B—C7—H7C	109.5	H16A—C16—H16B	109.5
O2—C8—H8A	109.5	O3—C16—H16C	109.5
O2—C8—H8B	109.5	H16A—C16—H16C	109.5
H8A—C8—H8B	109.5	H16B—C16—H16C	109.5
O2—C8—H8C	109.5		
			
C10—O1—C2—N1	−3.1 (3)	O1—C2—C1—O2	−2.0 (3)
C10—O1—C2—C1	177.2 (2)	N1—C2—C1—C6	−1.1 (4)
C2—O1—C10—N2	97.5 (3)	O1—C2—C1—C6	178.6 (2)
C2—O1—C10—C9	−88.9 (3)	N1—C4—C5—C6	0.0 (4)
C8—O2—C1—C6	6.7 (4)	C7—C4—C5—C6	178.7 (3)
C8—O2—C1—C2	−172.6 (2)	C1—C6—C5—C4	−0.5 (4)
C16—O3—C9—C10	−171.7 (2)	O3—C9—C14—C13	−178.9 (3)
C16—O3—C9—C14	7.9 (4)	C10—C9—C14—C13	0.7 (4)
C2—N1—C4—C5	0.0 (4)	C9—C10—N2—C12	1.1 (4)
C2—N1—C4—C7	−178.8 (2)	O1—C10—N2—C12	174.4 (2)
C4—N1—C2—O1	−179.1 (2)	N2—C10—C9—O3	178.5 (2)
C4—N1—C2—C1	0.6 (4)	O1—C10—C9—O3	5.4 (4)
C10—N2—C12—C13	−0.7 (4)	N2—C10—C9—C14	−1.1 (4)
C10—N2—C12—C15	179.8 (2)	O1—C10—C9—C14	−174.2 (2)
O2—C1—C6—C5	−178.4 (2)	N2—C12—C13—C14	0.3 (4)
C2—C1—C6—C5	1.0 (4)	C15—C12—C13—C14	179.8 (3)
N1—C2—C1—O2	178.3 (2)	C12—C13—C14—C9	−0.3 (4)

### Hydrogen-bond geometry (Å, °)

**Table:**

*D*—H···*A*	*D*—H	H···*A*	*D*···*A*	*D*—H···*A*
C6—H6···O3^i^	0.93	2.52	3.358 (3)	150

Symmetry codes: (i) −*x*+2, *y*+1/2, −*z*+1/2.
